# Pre-Exposure Gene Expression in Baboons with and without Pancytopenia after Radiation Exposure

**DOI:** 10.3390/ijms18030541

**Published:** 2017-03-02

**Authors:** Matthias Port, Francis Hérodin, Marco Valente, Michel Drouet, Reinhard Ullmann, Matthäus Majewski, Michael Abend

**Affiliations:** 1 Bundeswehr Institute of Radiobiology, 80937 Munich, Germany; MatthiasPort@bundeswehr.org (M.P.); reinhard1ullmann@bundeswehr.org (R.U.); matthaeusmajewski@bundeswehr.org (M.M.); 2 Institut de Recherche Biomedicale des Armees, 91220 Bretigny-sur-Orge, France; francis.herodin@wanadoo.fr (F.H.); marco.valente@live.fr (M.V.); michel.drouet@irba.fr (M.D.)

**Keywords:** radiosensitivity, pancytopenia, miRNA, gene expression, haematologic acute radiation syndrome (HARS)

## Abstract

Radiosensitivity differs in humans and likely among primates. The reasons are not well known. We examined pre-exposure gene expression in baboons (*n* = 17) who developed haematologic acute radiation syndrome (HARS) without pancytopenia or a more aggravated HARS with pancytopenia after irradiation. We evaluated gene expression in a two stage study design where stage I comprised a whole genome screen for messenger RNAs (mRNA) (microarray) and detection of 667 microRNAs (miRNA) (real-time quantitative polymerase chain reaction (qRT-PCR) platform). Twenty candidate mRNAs and nine miRNAs were selected for validation in stage II (qRT-PCR). None of the mRNA species could be confirmed during the validation step, but six of the nine selected candidate miRNA remained significantly different during validation. In particular, miR-425-5p (receiver operating characteristic = 0.98; *p* = 0.0003) showed nearly complete discrimination between HARS groups with and without pancytopenia. Target gene searches of miR-425-5p identified new potential mRNAs and associated biological processes linked with radiosensitivity. We found that one miRNA species examined in pre-exposure blood samples was associated with HARS characterized by pancytopenia and identified new target mRNAs that might reflect differences in radiosensitivity of irradiated normal tissue.

## 1. Introduction

Understanding individual human responses to radiation exposure with respect to tissue damage or developing radiation-related sequelae would be of benefit in several instances. For example, predictive strategies to determine the radiosensitivity of patient tumours and normal tissue a priori would be essential for personalized cancer therapy [1]. Astronauts traveling to Mars in 2030 would benefit from knowing their individualized radiation-related risk profile [2]; in radiological or nuclear scenarios, individuals exposed to the same magnitude of radiation might develop different degrees of haematologic acute radiation syndrome (HARS) because of inter-individual differences in radiosensitivity [3]. Identifying radiosensitive individuals would also aid in avoiding misclassification of those persons with low vs high exposures. 

Haematologic acute radiation syndrome develops days or weeks after radiation exposure depending on the absorbed dose. However, medical management decision making such as hospitalization or administration of granulocyte colony stimulating factor (G-CSF) would certainly benefit from an early diagnosis and prediction of the likely clinical course to help ensure a favourable outcome [4].

Haematologic acute radiation syndrome severity can be classified into low, moderate, severe, and lethal, according to the medical treatment protocols for radiation accident victims (METREPOL) [5]. From a clinical point of view, it would be preferable if patients who develop HARS might be distinguished from those who do not or do become pancytopenic.

We measured gene expression in pre-exposure peripheral blood samples of irradiated baboons that developed HARS with or without pancytopenia. We assumed the transcription status of the cells reflected differences in radiosensitivity. Many protein coding genes and microRNAs (miRNAs) have been previously identified that control biological processes such as cell proliferation, cell death, or DNA damage response and have also been associated with radiosensitivity [6,7,8]. Also, the established field of epigenetics examines the impact of environmental factors on the cellular response by modifications of the transcriptome through, for example, DNA methylation processes in the promotor region of the corresponding genes [9,10]. These differences in cellular activity are already linked with biological processes such as apoptosis, DNA repair, growth and differentiation which are known for their impact on radiosensitivity (reviewed in [11]). In collaboration with the French Army Biomedical Research Institute, we assessed blood samples obtained from baboons before partial/total body radiation exposure. The nonhuman primate models in the radiation research community are very valuable due to the close proximity of nonhuman primate and human response to radiation and treatment. The extended value of the model is its relation to the reality of a terrorist exposure or space travel where the nonuniform and heterogeneous exposure is relevant.

The blood cell counts (BCC) were measured in these baboons during the entire follow-up period in order to detect clinically significant HARS associated either with or without pancytopenia. Pancytopenia was defined as a reduced number of granulocytes (≤500/µL) over ≥10 days combined with a reduced number of thrombocytes (≤10,000/µL) measured at least once during the follow-up and a reduced number of erythrocytes corresponding to a haemoglobin (Hb) ≤8 g/dL measured at least once during the follow-up. On the blood samples taken before exposure, we performed a whole genome screening and identified protein coding messenger RNA (mRNA) genes associated with late occurring clinically relevant HARS with and without pancytopenia. These mRNAs were then validated using the gold standard—real-time quantitative polymerase chain reaction (qRT-PCR)—methodology for gene expression analysis. We also screened 667 miRNAs (stage I) using a qRT-PCR platform and the same samples. The selected candidate miRNAs were also validated on the remaining samples in stage II using the same qRT-PCR platform but restricting the analysis on the candidate miRNAs from stage I.

Since we are examining gene expression in blood samples before irradiation occurred we are trying to describe the activity status of the cells and its possible impact on the development of a more aggravated HARS, thus, reflecting differences in radiosensitivity. That differs from previously published work of our group where we successfully predicted the late occurring HARS based on gene expression changes measured at the first and second day after exposure and not on pre-exposure samples [12,13].

## 2. Results

### 2.1. Material Available for the Two Stage Study Design

Due to unusual blood cell counts before irradiation and sudden unexpected death after irradiation, one out of the 18 baboons had to be excluded, leaving 17 baboons eligible for analysis. 

**Figure 1:**
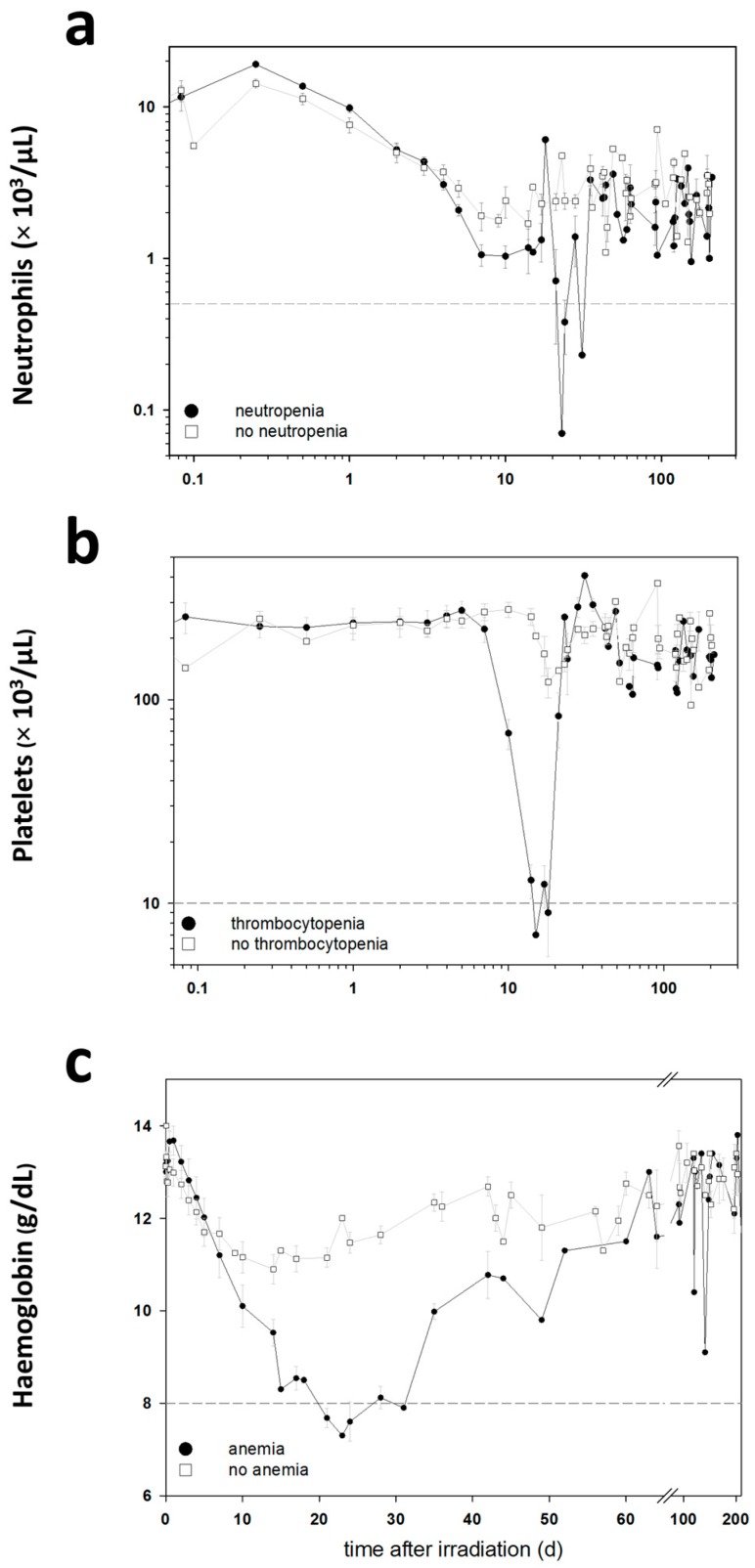
The mean change (±standard error of mean (SEM)) of neutrophils (**a**), platelets (**b**) and haemoglobin (**c**) in the blood cell counts of the baboons is displayed over the time, up to 203 days after irradiation. Black circles indicate the mean values for the animals with pancytopenia. White squares indicate the mean values of the animals without pancytopenia. The grey dashed lines show the thresholds for the definition of a pancytopenia (neutrophils 0.5 × 1000/µL; platelets, 10 × 1000/µL; haemoglobin, 8 g/dL). Measurements per time point range from *n* = 1 to *n* = 16.

During the screening approach, we assessed 10 whole genome microarrays for 10 blood samples collected before irradiation (Table 1). Five baboons developed HARS with pancytopenia and five blood samples were randomly selected from among those baboons who had HARS without pancytopenia (total *n* = 10) (Figure 1). The same blood samples were used for screening of 667 miRNAs with the commercially available low-density array (LDA) (Table 1).

For the validation of mRNA and miRNAs, we used the remaining seven blood samples. We performed the validation in two ways in which blood samples from the screening (total *n* = 10) were used and also on all available samples (HARS with pancytopenia, *n* = 5; HARS without pancytopenia, *n* = 12). Because only five baboons developed HARS with pancytopenia, sample size considerations necessitated use of the values from the screening stage in the validation stage.

### 2.2. Stage I: RNA Isolation Screening Results

From 2.5 mL whole blood, we isolated 9.8 µg total RNA on average before irradiation. RNA integrity (RIN) with a mean value of 8.4 (range 7.6–9.3) suggested high quality RNA sufficient for running both methods.

From about 20,000 protein coding mRNAs, 46% on average (range: 34%–54%) were expressed using our whole genome microarray data. Based on the fold-difference and the *p*-value, we selected candidate mRNAs for validation at stage II. We observed up to six-fold differences in differential gene expression (DGE) in blood samples from baboons who had HARS with pancytopenia relative to HARS without pancytopenia. According to these criteria, we selected 20 candidate mRNAs and forwarded them for validation in stage II using qRT-PCR.

During the screening of 667 miRNAs, we identified 9 miRNAs showing significant DGE in the HARS group with pancytopenia versus the group without pancytopenia.

### 2.3. Stage II: Validation Using Real-Time Quantitative Polymerase Chain Reaction Measurements

Twelve out of 20 candidate mRNAs developed an amplification plot in at least 60% of the samples and were eligible for analysis. Of them, four mRNAs showed a fold change (FC) ≥2. The heights of the FC were comparable to the FCs from the screening stage. However, all mean FCs were borderline or not significantly different (data not shown) between both HARS groups, so that none of the mRNAs from the screening stage could be validated.

**Figure 2:**
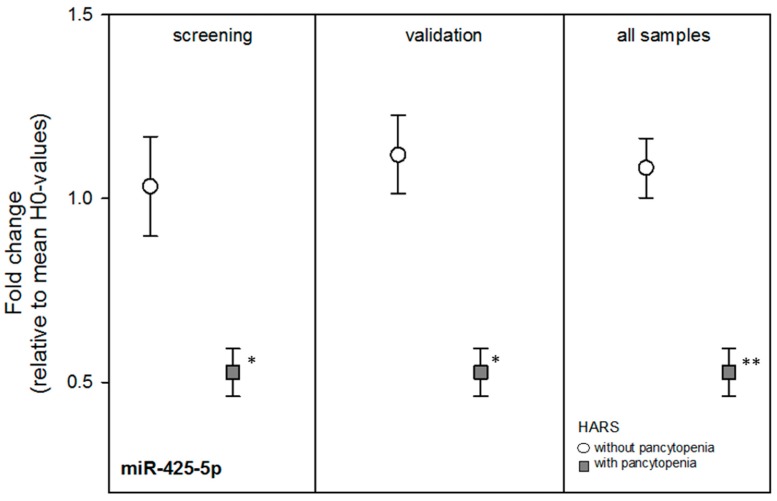
For graphical presentation of validated genes shown in Table 2, we plotted the mean fold change (FC) of miR-425-5p relative to the mean HARS 0 degree values (H0-values) separately for blood samples taken before irradiation in baboons developing HARS either with (squares) or without pancytopenia (circles). Error bars represent the standard error of the mean (SEM) and the numbers of blood samples examined are given in Table 2. Asterisks refer to *p*-values < 0.005 (*) or < 0.0005 (**). Mean FCs from the screening and the validation stage using ten and all 17 samples together are shown.

Nine miRNAs from the screening stage showed comparable mean FCs during the independent validation, but the mean FCs of the miRNAs were not significantly different by HARS group except for miR-425-5p (*p* = 0.002) (Table 2). Using all blood samples, most FCs among both HARS groups became borderline significant and miR-425-5p became more statistically significant (*p* = 0.0003) with about a two-fold DGE between both HARS groups (Table 2 and Figure 2). receiver-operator characteristic (ROC) analysis of miR-425-5p gene expression values indicated an almost complete separation (ROC = 0.98) of HARS groups.

### 2.4. Bioinformatic Analysis on Potential Target Genes (mRNAs) of miR-425-5p

Examination for potential target mRNAs of miR-425-5p revealed 282 entries using TargetScan [14] and 224 mRNAs were predicted with mirDataBase (miRDb, [15]). Both tools identified 69 mRNAs and were forwarded to PANTHER [16,17]. The number of genes regulating macromolecular metabolic processes (*p* = 2 × 10^−5^) and gene expression (*p* = 2.6 × 10^−4^) showed approximately two-fold overrepresentation. These changes were significantly associated with an about two-fold overrepresentation in the number of genes coding for intracellular membrane bound organelles (*p* = 4.5 × 10^−6^).

**Figure 3:**
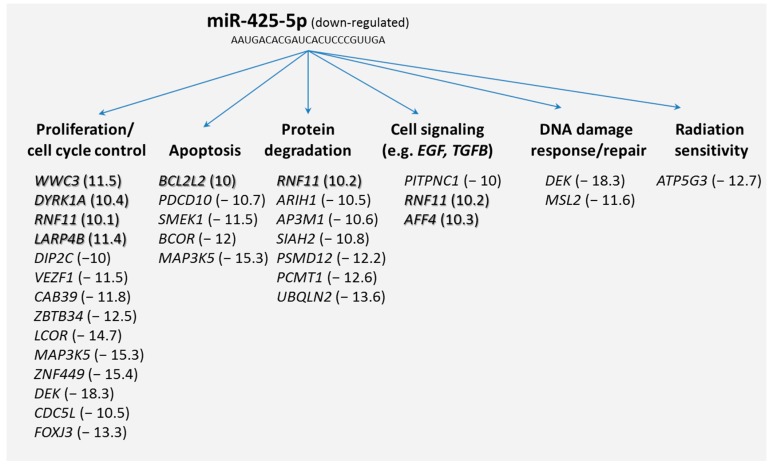
Potential target genes of miR-425-5p that were >two-fold up (shadowed) or downregulated between the HARS groups were grouped according to their coding function using their gene ontology data (The National Center for Biotechnology Information (NCBI) data base). Fold changes are given in parentheses.

Secondly, we screened our microarray data and from the potential 69 mRNAs we selected those target genes that were differentially expressed (≥two-fold difference) among both HARS groups. It appeared that 43 mRNAs were either up-regulated (*n* = 7) or downregulated (*n* = 36). Grouping these target mRNAs according to their annotations (gene ontology) showed that many of the differentially expressed genes coded for biological processes such as proliferation, apoptosis, cell signaling (e.g., epidermal growth factor (*EGF*)- or transforming growth factor-β (*TGFB*) signaling pathway), DNA damage response/repair, and radiation sensitivity (Figure 3). 

## 3. Discussion

Gene expression activity is known to provide hints toward differences in radiosensitivity and miRNAs are currently under intensive observation [6,7]. We examined the transcriptional (protein coding mRNAs) and post-transcriptional (miRNA) activity in pre-exposure peripheral blood samples of baboons that developed HARS with or without pancytopenia after irradiation. HARS with pancytopenia represents a more aggravated disease and was observed after equivalent whole body exposures with 2.5 or 5 Gy (Table 1). Interestingly, two out of the five baboons developing a more aggravated HARS (with pancytopenia) were exposed to half of the dose (2.5 Gy) than most of the animals that were exposed to 5 Gy and that subsequently developing a less aggravated HARS. So dose seems to be not the major predictor of HARS with or without pancytopenia but other factors related to inherent radiosensitivity might play a role.

During the screening approach we identified 20 mRNAs and nine miRNAs. During the validation stage, none of the mRNAs but most of the miRNA species revealed statistically significant or borderline significant gene expression differences between HARS groups with pancytopenia or without pancytopenia. In particular miR-425-5p appeared promising and the ROC (0.98) indicated an almost complete separation of both HARS groups. 

In tumor cells, miRNAs are associated with radiosensitivity through their regulatory control of biological processes such as DNA damage response/repair, cell cycle checkpoints, regulation of reactive oxygen species, survival pathways, cancer stem cells and drug resistance [6,7,18,19]. Less is known about how miRNAs contribute to normal tissue radiation response (reviewed in [7]). We found that miR-425-5p appeared promising with respect to normal tissue response to radiation in our examination of healthy baboons. Accordingly, this miRNA has been previously associated with chemo- and radiosensitivity [20,21], cell proliferation [22,23], and cell death [20].

Our results pointed to understanding potential mRNA target genes of miR-425-5p. We used two different databases, for which 69 mRNA species were identified and PANTHER indicated genes that were involved in regulating macromolecular metabolic processes, gene expression, and coding for intracellular membrane bound organelles. Searching for differentially expressed mRNAs, 43 out of the 69 potential target mRNAs were identified taking advantage of our whole genome screening approach using microarrays. These genes coded for biological processes such as proliferation, apoptosis, protein degradation, cell signaling, DNA damage response/repair, and radiation sensitivity (Figure 3). Since we found that miR-425-5p appeared down-regulated, we also focused on up-regulated mRNAs (*n* = 7) assuming a direct and inverse regulatory effect of the miR-425-5p on the target gene (so called first neighbor). Along this line of reasoning, we could include biological processes such as proliferation, apoptosis, protein degradation, and cell signaling. All these biological processes, with the exception of protein degradation, are known to be linked with radiosensitivity (see above). We further detected mRNAs not related to these expected biological processes. Such genes were those coding for the cholesterol metabolism (*HDLBP*), heat shock protein (*HSPH1*), mRNA metabolism, and transport related genes (heterogeneous nuclear ribonucleoprotein (hnRNP)-family, *SYNCRIP*) or pre-mRNA processing genes (*SRSF11*) as well as mRNAs linked to the Wnt–β-catenin signaling pathway (*ARMCX3*) or to tumorigenesis (*CPEB2*, *FAM133B*, *KLF3*). Interestingly, in previous work we had identified *HNRPA1* and *WNT3* as radiation-induced genes. *HNRPA1*, belonging to the hnRNP-family, appeared to be involved in atherosclerotic processes in Mayak workers with internal plutonium exposure [24]. *WNT3* was identified as a robust bioindicator that predicted more severe HARS in the first two days after exposure in baboons [12].

Noteworthy and as already stated, due to unusual blood cell counts before irradiation and sudden unexpected death after irradiation, one out of the 18 baboons had to be excluded from analysis. However, the dramatic reduction in blood cell counts observed in this baboon could be linked with increased radiosensitivity. An additional sensitivity analysis revealed a miR-425-5p RNA-equivalent value (CT-value) of 8.4 in the pre-exposure blood sample of this baboon (data not shown), which fits to the expected mean CT-value of 8.1 associated to the HARS group comprising a pancytopenia (Table 2). This could be interpreted as an indicator for increased radiosensitivity in the baboon excluded from analysis and might explain the dramatic decrease in blood cell counts after exposure.

We also examined the miR-425-5p expression at 1, 2, 7, 28, and 90 days after exposure (data not shown). Interestingly, miR-425-5p seemed to be marginally altered by radiation exposure (mean: 8.7; standard error of mean (SEM): 0.8, RIN ranged from 7.6–9.3). This might have diagnostic implications, since miR-425-5p measurements after radiation exposure could provide evidence for a more aggravated HARS developing in individuals exposed to doses where no severe HARS would be expected. 

Some limitations of our study should be kept in mind. We performed gene expression measurements on microarrays and qRT-PCR using human genomic sequences because the baboon transcriptome was not publically available. Given the high homology of both genomes (93%) and previously reports by other groups [25,26,27], we proceeded as described above. Since we used TaqMan chemistry with human primer and probe sequences (high sensitivity and specificity), it is more likely that we lost some of the detectable human RNA species due to a mismatch with the baboon genome, rather than producing false positives.

The small sample size of our study represents a weakness and surely reduced the number of successfully validated candidate genes. Given the small sample size we cannot exclude the possibility of our results occurring by chance. However, the miR-425-5p *p*-values corrected for multiple comparison using the most stringent Bonferroni approach remained significant. That from the statistical point of view is an objective parameter assuming an association. In previous work on this baboon model we already searched for gene expression changes associated with other endpoints [12,13]. Preliminary validation steps using in vivo irradiated human patient samples confirmed most of our potential gene targets. These results stimulate us to proceed with our research on miR-425-5p. However, future work certainly will consider larger sample sizes and results will be validated with additional species 

Although limited in size animal studies such as ours are urgently needed in order to provide potential biological indicator of e.g., radiosensitivity with strong implication for use in humans due to the close proximity of nonhuman primates with humans. 

In summary, we identified miR-425-5p in pre-exposure blood samples as a promising biomarker of radiosensitivity in baboons that developed a more aggravated form of the HARS after exposure. We could also identify new potential mRNA target genes and associated biological processes, presumably associated with radiosensitivity. This research adds to the few reports on radiosensitivity of normal tissue.

## 4. Materials and Methods

### 4.1. Animals

Eighteen baboons were bred by the Centre National de la Recherche Scientifique (Rousset sur Arc, France) for the purpose of biomedical research. In the nonhuman primate facility of the French Army Biomedical Research Institute, the baboons were placed in individual cages at 21 °C, with a relative humidity of 55% and a 12 h/12 h light/dark schedule. The animals received fresh fruit and solid food twice a day, and had access to water ad libitum. The average age of the male baboons was 8.1 years (±3.3 years) and weighed 23.7 ± 5.2 kg. The experiment was approved by the French Army Animal Ethics Committee (No 2010/12.0). All baboons were treated in compliance with the European legislation related to animal care and protection in order to minimize pain and damage. The total number of baboons evaluated in this study decreased to 17, for reasons described below.

### 4.2. Irradiation

The animals were anesthetized with a combination of tiletamine and zolazepam (6 mg·kg^−1^ intramuscularly, Zoletil 100, Virbac, Carros, France) before irradiation. Then, the baboons were placed in restraint chairs, sitting orthogonally, front to a horizontal and homogeneous field of gamma rays delivered by a ^60^Co source (IRDI 4000, Alsthom, Levallois, France) to perform either total body irradiation (TBI) or partial body irradiation (PBI, Table 1). In order to attain different patterns of PBI, a 20-cm thick lead screen was used to shield different parts of the body as detailed in Table 1. Two baboons were exposed to 5 Gy TBI and two others to 2.5 Gy TBI. Eight different exposure patterns were simulated and two baboons were exposed per pattern which summed up to 16 baboons receiving PBI (for details see [28]) corresponding to an equivalent TBI dose of 2.5 or 5 Gy (Table 1). Two dose rates were used (8 cGy/min for 5 Gy TBI and 5 Gy 50% PBI, and 32 cGy/min for all other situations) because the Cobalt 60 source was changed during this study. Moreover, to achieve the same homogeneous radiation field whatever the dose rate, all baboons were irradiated at the same distance from the source. Consequently, radiation exposures lasted between 8 min and 62 min. The mid-line tissue (right anterior iliac crest) dose in air was measured with an ionization chamber. Delivered doses were controlled by alumina powder thermoluminescent dosimeters placed on different cutaneous areas (thorax, thoracic and lumbar vertebrae, head, tibia, femur, femoral head, for details see [28]). The different exposure patterns chosen appeared in particular of significance when considering terrorist exposure or space travel where the nonuniform and heterogeneous exposure is relevant.

### 4.3. Blood Collection, Determination of Haematologic Acute Radiation Syndrome Severity Scores and Pancytopenia

Using changes in BCC, the severity scores (0: unexposed; 1–4: low-severe degree) of HARS was determined following METREPOL [5]. Pancytopenia was identified based on a reduced number of granulocytes (≤500/µL over ≥10 days), thrombocytes (≤10,000/µL) and erythrocytes (Hb ≤ 8 g/dL). Reduced numbers of thrombocytes and erythrocytes had to be measured at least once during the follow-up. The HARS score as well as pancytopenia was based on changes in differential blood counts taken at up to 22 time points over the course of 7–203 days after exposure (Table 1). Whole blood samples for gene expression measurements were taken only before irradiation (0 h) and were assessed within the two groups of baboons who developed HARS with our without pancytopenia. 

### 4.4. RNA Extraction and Quality Control

Whole blood samples (2.5 mL) were processed following the PAXgene Blood RNA system (BD Diagnostics, PreAnalytiX GmbH, Hombrechtikon, Switzerland). In brief, blood was drawn into a PAXgene Blood RNA tube at the French Army Biomedical Research Institute. The tube was gently inverted (10 times), and then stored at room temperature overnight at −20°. After all samples were collected, the PAXgene tubes were sent to Germany for further processing. After thawing, washing, and centrifugation, cells in the supernatant were lysed (Proteinase K, BD Diagnostics, PreAnalytiX GmbH, Hombrechtikon, Switzerland) followed by addition of Lysis/Binding Solution taken from the mirVana Kit (Life Technologies, Darmstadt, Germany). With the mirVana kit, total RNA, including small RNA species, was isolated by combining a Phenol-Chloroform RNA precipitation with further processing using a silica membrane. After several washing procedures DNA residuals became digested on the membrane (RNAse-free DNAse Set, Qiagen, Hilden, Germany). RNA was eluted in a collection tube and frozen at −80 °C. Quality and quantity of isolated total RNA were measured spectrophotometrically (NanoDrop, PeqLab Biotechnology, Erlangen, Germany). RNA integrity was assessed by the 2100 Agilent Bioanalyser (Life Science Group, Penzberg, Germany) and DNA contamination was controlled by conventional PCR using an actin primer. We used only RNA specimens with a ratio of A_260_/A_280_ ≥2.0 (Nanodrop) and RNA integrity number (RIN) ≥7.5 for whole genome microarray (IMGM Laboratories, Martinsried, Germany) or RIN ≥7.3 for qRT-PCR analyses.

### 4.5. Stage I Screening

Whole genome screening for differentially expressed genes (protein coding mRNAs) was performed on 10 RNA samples (baboons with HARS and absence or presence of pancytopenia *n* = 2 × 5, Table 1). We used the Agilent oligo microarray GE 8 × 60 K (Agilent Technologies, Waldbronn, Germany) combined with a one-color based hybridization protocol of GeneSpring GX12 software for data analysis as described in detail elsewhere [29]. We analyzed gene expression by quantile normalized log_2_-transformed probe signals as an outcome. We used the nonparametric Mann Whitney (MW) test to compare gene expression in the HARS with pancytopenia group considering the HARS without pancytopenia as the reference group. Only those gene transcripts that had a call “present” in at least 60% of RNA specimens were included in the analysis of gene expression and only genes with MW *p*-values ≤0.05 and with a ≥two-fold gene expression difference among compared groups were considered to represent a candidate gene for validation in stage II. Due to the explorative nature of this study, the low sample size and the nonparametric statistic employed, we did not correct for multiple comparisons on the screening stage I of the study. We considered multiple comparisons in the bioinformatic approach as well as the validation stage II of our study where the numbers of hypothesis tested in parallel was reduced from about 20,000 (stage I) to 20 mRNAs and nine miRNAs in stage II (see below). Gene expression data presented in this publication have been deposited at the NCBI’s Gene Expression Omnibus (GEO accession number GSE77254).

Screening for differentially expressed miRNAs using qRT-PCR was performed on the same 10 RNA samples used for the whole genome mRNA screening. Methodological details are shown in the following paragraph.

### 4.6. Stage II: Validation of Stage I Candidate Genes via qRT-PCR

We validated the mRNA candidate genes from stage I (screening) using the remaining RNA samples (Table 1). We used a custom low density array (LDA high throughput qRT-PCR platform) and TaqMan chemistry. A 1 µg RNA aliquot of each RNA sample was reverse transcribed using a two-step PCR protocol (High Capacity Kit). 400 µL cDNA (1 µg RNA equivalent) and was mixed with 400 µL 2× RT-PCR master mix and pipetted into the 8 fill ports of the LDA. Cards were centrifuged twice (1200 rpm, 1 min, Multifuge3S-R, Heraeus, Germany), sealed, and transferred into the 7900 qRT-PCR instrument. The qRT-PCR was processed for two hours following the qRT-PCR protocol for 384-well LDA format. All measurements were run in duplicate. 

A commercially available 384-well LDA was used that provided simultaneous detection of 380 different miRNA species. Two different LDAs (type A and B) were combined so that the detection of 667 miRNA species (partly spotted in duplicate to completely fill the LDA) was possible. Aliquots from each RNA sample (in general 2 µg total RNA/LDA type A/B) were reversely transcribed without preamplification over three hours using “*Megaplex pools without preamplification l for microRNA expression analysis protocol*.” Using different sets of primers, two kinds of cDNAs suitable for each of both LDAs were created. In a second step, the whole template cDNA and 450 µL 2× qRT-PCR master mix were adjusted to a total volume of 900 µL by adding nuclease free water, and aliquots of 100 µL were pipetted into each fill port of a 384-well human LDA. Cards were centrifuged twice (see above), sealed, transferred into the 7900 qRT-PCR instrument and again the 384-well LDA qRT-PCR protocol was run over two hours.

All technical procedures for qRT-PCR were performed in accordance with standard operating procedures implemented in our laboratory in 2008 when the Bundeswehr Institute of Radiobiology became certified according to DIN EN ISO 9001/2008. All chemicals for qRT-PCR using TaqMan chemistry were provided by Life Technologies, Darmstadt, Germany.

For the custom LDA CT values (threshold cycles) were normalized relative to the 18S rRNA measured in an aliquot of the RNA samples using a 96 well format TaqMan qRT-PCR platform. We have found that this approach to normalization was more robust compared to the use of the internal control (glyceraldehyde*-*3*-*phosphate dehydrogenase (GAPDH) and 18S rRNA) spotted on the LDA. For the commercial LDA we used the median miRNA expression on each LDA for normalization purposes, because this proved to be the more robust and slightly more precise method compared to a normalization approach using a housekeeping miRNA species provided on the LDA (data not shown). The CT-values of the housekeeping gene was subtracted from the CT-value of each of the spotted genes, following the ∆CT−quantitative approach for normalization purposes. 

### 4.7. Bioinformatics

We identified potential target genes of miR-425-5p using the overlapping number of mRNAs detected with the mirDataBase (miRDb, [15]) and TargetScan [14]. For these potential target genes we performed gene set enrichment analyses using PANTHER pathway software version 11.1 [16,17]. PANTHER groups genes with similar biological function based on their annotation (reference list was the current homo sapiens gene ontology database). The *p*-values were corrected for multiple testing employing the Bonferroni algorithm. In a second approach, we used our whole genome microarray data and examined whether the predicted target genes (mRNAs) were truly differentially expressed (≥two-fold and detected in ≥60% of the samples per HARS group). These mRNAs were finally grouped according to the biological processes for which they were coding.

### 4.8. Statistical Analysis

Using quantitative qRT-PCR gene expression results, we examined HARS groups with and without pancytopenia. Descriptive statistics (*n*, mean, standard deviation, min, max) and *p*-values (Student’s *t*-test) were calculated for each of the variables (candidate mRNAs and miRNAs) and per time point. We assessed the assumptions of normality (Kolmogorov-Smirnov) and equal variance and if required utilized either the pooled (equal variance) or the Satterthwaite variant of the Student’s *t*-test (unequal variance). Unconditional logistic regression analysis was performed for each of the independent variables (genes) of interest separately (univariate) on the binary outcome variable of HARS group with or without pancytopenia. Using logistic regression we also determined the area under a receiver-operator characteristic (ROC) curve providing a reasonable indication of overall diagnostic accuracy. ROC areas of 1.0 indicate complete agreement between the predictive model and the HARS group and thus a clear distinction between baboons’ subsequently showing clinically relevant HARS with or without pancytopenia. All calculations were performed using SAS (release 9.2, Cary NC, USA).

## Figures and Tables

**Table 1:** Radiation exposure scenarios and resulting haematologic acute radiation syndrome severities.

Experimental Design		Exposure			HARS Degree
	ID No.	Radiation Dose (Gy Midline Tissue Free in Air Dose)	Equivalent Whole Body Dose (Gy)	Exposure Details	
**Screening**					
*HARS without pancytopenia*					
	1	6.25 PBI 80%	5	two legs shielded	2
	2	10 PBI 50%	5	left hemibody exposed	1–2
	3	5 TBI	5	TBI	2
	4	7.5/2.5 TBI	5	TBI	3
	5	5.55 PBI 90%	5	one leg shielded	2
*HARS with pancytopenia*					
	6	6.25 PBI 80%	5	two legs shielded	2
	7	6.25 PBI 80%	5	head neck shielded	2
	8	2.5 TBI	2.5	TBI	2–3
	9	2.5 TBI	2.5	TBI	2–3
	10	5 TBI	5	TBI	2
**Validation**					
*HARS without pancytopenia*					
	1	5 PBI 50%	2.5	left hemibody exposed	2
	2	5 PBI 50%	2.5	left hemibody exposed	2
	3	15 PBI 30%	5	head + arms exposed	1–2
	4	15 PBI 30%	5	head + arms exposed	1–2
	5	6.25 PBI 80%	5	two legs shielded	2–3
	6	5.55 PBI 90%	5	one leg shielded	3
	7	10 PBI 50%	5	left hemibody exposed	1–2

The table summarizes the radiation exposure scenarios and resulting haematologic acute radiation syndrome (HARS) severities for baboons with pre-radiation blood samples. For the screening stage, five blood samples were assessed for two HARS groups (with and without pancytopenia). The validation was performed on the remaining blood samples (*n* = 7) from the HARS group that developed HARS without pancytopenia. Partial (PBI) and total body exposures (TBI) were performed and exposure details are shown in the table. The exposure pattern called 7.5/2.5 Gy TBI represents a sequential protocol of irradiation using 2.5 Gy TBI at first followed by an additional 5 Gy exposure with hemibody shielding, so that the unshielded half of the two irradiated baboons received an additional 5 Gy exposure. Exposure between the two fractions was stopped for 5 min. The radiation dose is given as the midline tissue free in air dose measured on the right anterior iliac crest.

**Table 2:** Real-time quantitative polymerase chain reaction results.

miRNA Species	HARS without Pancytopenia					HARS with Pancytopenia						
	No. Samples Evaluated	Mean	SD	Min.	Max.	No. Samples Evaluated	Mean	SD	Min.	Max.	FC	Student’s *t*-test
**screening**												
miR-425	5	7.2	0.4	6.5	7.5	4	8.1	0.3	7.7	8.4	0.5	0.005
miR-192	5	12.7	0.4	12.4	13.2	4	14.2	0.9	13.1	15.1	0.4	0.01
miR-149-raute	5	14.4	0.5	13.8	15.0	4	13.4	0.5	12.9	14.0	2.0	0.02
miR-598	3	19.5	0.1	19.4	19.7	2	19.0	0.0	19.0	19.1	1.4	0.02
miR-30c	5	5.3	0.7	4.2	5.8	4	6.7	0.9	5.6	7.6	0.4	0.03
miR-1290	5	17.2	0.7	16.5	18.1	4	16.1	0.4	15.7	16.5	2.1	0.03
miR-15b-raute	5	8.8	0.8	8.1	10.2	4	10.3	1.0	9.6	11.7	0.4	0.04
miR-222	5	6.9	0.5	6.3	7.6	4	7.7	0.4	7.3	8.1	0.6	0.04
miR-942	5	8.7	0.6	8.0	9.5	4	9.9	0.9	9.0	11.2	0.4	0.05
**validation**												
miR-425	7	7.1	0.4	6.6	7.9	4	8.1	0.3	7.7	8.4	0.5	0.002 *
miR-192	7	13.1	0.9	12.2	14.9	4	14.2	0.9	13.1	15.1	0.5	0.09
miR-149-raute	7	13.4	0.5	12.7	14.2	4	13.4	0.5	12.9	14.0	1.0	0.9
miR-598	5	19.2	0.9	18.1	20.2	2	19.0	0.0	19.0	19.1	1.1	0.7
miR-30c	7	5.8	0.9	4.6	7.0	4	6.7	0.9	5.6	7.6	0.5	0.1
miR-1290	7	16.6	0.4	16.0	17.1	4	16.1	0.4	15.7	16.5	1.3	0.2
miR-15b-raute	7	9.2	1.1	8.0	11.4	4	10.3	1.0	9.6	11.7	0.5	0.1
miR-222	7	7.3	1.1	5.7	9.2	4	7.7	0.4	7.3	8.1	0.7	0.4
miR-942	7	9.3	0.7	8.3	10.2	4	9.9	0.9	9.0	11.2	0.6	0.2
**validation on all samples**												
miR-425	12	7.1	0.4	6.5	7.9	4	8.1	0.3	7.7	8.4	0.5	0.0003 *
miR-192	12	13.0	0.7	12.2	14.9	4	14.2	0.9	13.1	15.1	0.4	0.02
miR-149-raute	12	13.8	0.7	12.7	15.0	4	13.4	0.5	12.9	14.0	1.3	0.3
miR-598	8	19.3	0.7	18.1	20.2	2	19.0	0.0	19.0	19.1	1.2	0.6
miR-30c	12	5.6	0.8	4.2	7.0	4	6.7	0.9	5.6	7.6	0.5	0.03
miR-1290	12	16.8	0.7	16.0	18.1	4	16.1	0.4	15.7	16.5	1.6	0.07
miR-15b-raute	12	9.0	1.0	8.0	11.4	4	10.3	1.0	9.6	11.7	0.4	0.04
miR-222	12	7.1	0.9	5.7	9.2	4	7.7	0.4	7.3	8.1	0.7	0.2
miR-942	12	9.1	0.7	8.0	10.2	4	9.9	0.9	9.0	11.2	0.5	0.06

The table summarizes real-time quantitative polymerase chain reaction (qRT-PCR) results (normalized threshold cycle (CT)-values) of microRNAs (miRNAs) examined during the screening and validation (remaining samples and with all samples combined) stage. Descriptive statistics reflect the distribution and the fold change (FC) difference among miRNAs differentially expressed relative to mean HARS 0 severity degree (H0) as the reference and between both haematologic acute radiation syndrome (HARS) groups in order to compare discrimination between HARS without and with pancytopenia. Numbers for columns entitled “No. Samples Evaluated” reflect the total number of blood sample examined per group. Depending on the miRNA this number can be smaller. The Student’s *t*-test was performed for comparison of mean FC between the two HARS groups. *p*-values corrected for multiple comparisons (Bonferroni) are marked with an asterisk when they reached values of *p* < 0.006 (*p* = 0.05/9 genes/hypothesis). SD: Standard deviation.
